# Identification of putative olfactory G-protein coupled receptors in Crown-of-Thorns starfish, *Acanthaster planci*

**DOI:** 10.1186/s12864-017-3793-4

**Published:** 2017-05-23

**Authors:** Rebecca E. Roberts, Cherie A. Motti, Kenneth W. Baughman, Noriyuki Satoh, Michael R. Hall, Scott F. Cummins

**Affiliations:** 1 Genecology Research Centre, Faculty of Science, Health, Education and Engineering, University of the Sunshine Coast, Maroochydore DC, QLD 4558 Australia; 2 Australian Institute of Marine Science (AIMS), Cape Ferguson, Townsville, QLD 4810 Australia; 3 Marine Genomics Unit, Okinawa Institute of Science and Technology Graduate University, Onna, Okinawa 904-0495 Japan

**Keywords:** Olfaction, GPCR, In situ hybridisation, Starfish, COTS

## Abstract

**Background:**

In marine organisms, and in particular for benthic invertebrates including echinoderms, olfaction is a dominant sense with chemosensation being a critical signalling process. Until recently natural product chemistry was the primary investigative approach to elucidate the nature of chemical signals but advances in genomics and transcriptomics over the last decade have facilitated breakthroughs in understanding not only the chemistry but also the molecular mechanisms underpinning chemosensation in aquatic environments. Integration of these approaches has the potential to reveal the fundamental elements influencing community structure of benthic ecosystems as chemical signalling modulates intra- and inter-species interactions. Such knowledge also offers avenues for potential development of novel biological control methods for pest species such as the predatory Crown-of-Thorns starfish (COTS), *Acanthaster planci* which are the primary biological cause of coral cover loss in the Indo-Pacific.

**Results:**

In this study, we have analysed the COTS sensory organs through histological and electron microscopy. We then investigated key elements of the COTS molecular olfactory toolkit, the putative olfactory rhodopsin-like G protein-protein receptors (GPCRs) within its genome and olfactory organ transcriptomes. Many of the identified *Acanthaster planci* olfactory receptors (*ApORs*) genes were found to cluster within the COTS genome, indicating rapid evolution and replication from an ancestral olfactory GPCR sequence. Tube feet and terminal sensory tentacles contain the highest proportion of *ApORs*. In situ hybridisation confirmed the presence of four *ApORs*, *ApOR15, 18, 25 and 43* within COTS sensory organs, however expression of these genes was not specific to the adhesive epidermis, but also within the nerve plexus of tube feet stems and within the myomesothelium. G alpha subunit proteins were also identified in the sensory organs, and we report the spatial localisation of Gαi within the tube foot and sensory tentacle.

**Conclusions:**

We have identified putative COTS olfactory receptors that localise to sensory organs. These results provide a basis for future studies that may enable the development of a biological control not only for COTS, but also other native pest or invasive starfish.

**Electronic supplementary material:**

The online version of this article (doi:10.1186/s12864-017-3793-4) contains supplementary material, which is available to authorized users.

## Background

Chemosensation is the primary sense used by aquatic organisms in which visual or acoustic signalling may be limited. Chemical signalling can operate over short and long distances in aquatic environments and organisms modulate behaviour and modify gene expression in response to these external chemical signals [1]. These chemical signals must bind to a receptor to trigger a physiological and/or behavioural response in the organism and include pheromones and general odorants. An analysis of the molecular components of the chemosensory system is critical to our understanding of how organisms respond, either positively, (i.e. attraction) or negatively, (i.e. repulsion) to chemical signals in the environment.

Olfactory receptors, such as the olfactory G protein-coupled receptors (GPCRs), include a family of seven-transmembrane (7TM) receptors that bind extracellular molecules. They are often the largest and most diverse protein family within animal genomes, highlighting the significance of the fundamental ability of animals to discriminate between chemical stimuli [2]. For example, over 1 per cent of the total protein-coding genome of *Homo sapiens* encodes GPCRs [3]. At the genome level, a characteristic feature of olfactory GPCRs is that they are often found in tandem arrays as a result of large-scale gene duplication and rapid gene evolution [4]; this results in enormous diversity between phyla [2]. Olfactory-specific GPCRs are typically expressed in the sensory epithelia of specialized organs, such as the vomeronasal organ in vertebrates, the rhinophore in sea slugs, or the antennae of insects [5–7]. Vertebrates typically possess vomeronasal receptors such as V1R and V2R gene families within the GPCR superfamily, which differ considerably from the olfactory receptors found in invertebrates [2]. Insect olfactory receptors display seven-transmembrane structure, however an inverted topology results in a lack of sequence similarity to those found in vertebrates and the inability to couple with G proteins for signal transduction [8, 9]. GPCRs in other invertebrate phyla have undergone frequent lineage-specific expansions throughout evolutionary history and hence bear little similarity to those found in vertebrates [2]. Upon activation, GPCRs activate intracellular signal transduction pathways which may lead to a physiological and behavioural response [10–12]. Signal transduction is achieved through G proteins, many of which are highly conserved across animal phyla [13]. G proteins act as heterotrimeric complexes consisting of a primary alpha subunit which activates the closely associated beta and gamma subunits [14]. There are four main families of G α proteins - Gαi, Gαs, Gαq and Gα12 - which trigger different effectors, including phospholipase, adenylyl cyclase and ion channel signalling pathways [14]. These four main families have diversified in many phyla and are known to contain several subfamilies: Gαs includes the Gαolf subfamily, which are known to be involved in signal transduction specifically from olfactory receptors in vertebrates; Gαi contains subfamilies Gαo, Gαt and Gαz; Gαq contains subfamilies Gα11, Gα14, Gα15 and Gα16; and Gα12 also contains the subfamily Gα13 [15–17].

Olfaction through GPCR signalling is critical for all organisms and arose early in evolutionary history; unicellular bacteria are known to coordinate group-based behaviours and regulate gene expression through the release of signalling molecules, such as homoserine lactones, which bind to GPCRs [18]. Since their discovery, chemosensory GPCRs have been found across the animal and plant kingdoms, from invertebrates such as the nematode worm *Caenorhabditis elegans* (which has a larger GPCR repertoire, as a per cent of its genome, than any other animal investigated) [19], to vertebrates including humans [20]. New insights into the molecular basis of olfaction in aquatic invertebrates have been recently reported [21, 22] This has primarily been due to advances in genomics [23]; for example, sequencing of the purple sea urchin *Strongylocentrotus purpuratus* genome [24] enabled the *in silico* identification of over 900 rhodopsin-like GPCRs, including a novel family of independently-expanded olfactory receptors, the *surreal*-GPCRs (Sea URchin Rapidly ExpAnded Lineages of GPCRs) [21]. This research was the first to reveal the molecular components of olfaction in the echinoderm phyla.

Olfaction is essential in aquatic environments, particularly for invertebrates such as echinoderms. With the exception of mechanoreception and light-sensing eyespots, these animals lack other well-developed senses. As such, echinoderms rely heavily on olfaction throughout their life cycle, including the identification of suitable habitat, detecting food, sensing predators and synchronisation of reproductive behaviours such as aggregations, mass spawning events and larval settlement and metamorphosis [25–30]. Similar to other echinoderms, the Crown-of-Thorns starfish (COTS; *Acanthaster planci*) are not known to have any acoustic sense and vision, although well-developed, only operates over distances of a few metres [30]. In contrast, chemical signalling can operate over long distances and olfaction is the primary sense which regulates many aspects of their life cycle. Mechanoreception is also used, whereby non-GPCR receptors provide information to the nervous system about touch, pressure and vibrations [31].

All echinoderms possess epidermis-covered coelomic projections known as tube feet, which are connected to the water vascular system and function in feeding, locomotion, gas exchange, waste diffusion and attachment to substratum [32]. As do all Asteroids in the Velatid, Forcipulatid and Spinulosid families, COTS have reinforced disc-ending tube feet, consisting of a basal cylindrical stem topped by a flattened disc [32]. However, not all COTS tube feet have the same morphology. Well-characterised in other Asteroid species in earlier studies [33, 34], the anatomy of these organs consists of three tissue layers: a connective tissue layer between the inner and outer epithilia. Several knob-ending tube feet, in which the distal part is pointed, can be found at the tip of each arm and have been termed terminal sensory tentacles. As their morphology is different, the two types may serve different functions [33]. Histological studies and evidence from transmission-electron microscopy indicate that cells assumed to be chemo- or mechano- sensory are abundant within the adhesive epidermis of the tube feet and sensory tentacles in starfish [32–34]. An epineural nerve plexus is discernible beneath the epidermis, thickened on one side of the transverse sections to allow formation of the longitudinal nerve and usually in areas in which secretory and presumed sensory cells are found [34].

Echinoderms respond to a variety of environmental stimuli [27–29, 35]. The tube feet of sea urchins and starfish have been observed to respond to chemical cues, a result substantiated by the relative abundance of rhodopsin-like GPCRs expressed in these tissues [21]. Based on these lines of evidence, these tissues may act as sensory organs in COTS. COTS show similar avoidance behaviours to sea urchins when exposed to eluate containing secretions from one of their main predators, the giant triton, *Charonia tritonis* [36]. COTS are also thought to use olfactory mechanisms when forming aggregations just prior to synchronous spawning events [25, 26]. The identification of key molecular elements of the olfactory system would enable for manipulation of such behaviours. Similar studies have recently investigated olfactory receptors as targets for biological control in moths, which are pests affecting many horticultural crops [37]. The first draft genome of a starfish, COTS, has recently been published and has enabled the investigation of olfaction in this species. With a genome size of approximately 421 Mb, and 24,323 genes, there is a large family of 775 rhodopsin-like GPCRs, constituting >3% of the total protein-coding genome [38].

In this study we have applied histological, molecular and genomic bioinformatic techniques to: (i) investigate the structure and morphology of the COTS olfactory organs; (ii) identify putative olfactory GPCRs in COTS; (iii) determine the spatial expression of some putative olfactory GPCRs within their olfactory organs; and (iv) investigate the G proteins involved in signal transduction from GPCRs. Given the position of starfish among the earliest deuterostomes, studying chemoreception in this group will provide novel insights into the evolution of olfactory systems, as well as identifying GPCRs which will be potential targets for next-generation control technology in COTS.

## Methods

### Animals and tissue collection

Adult COTS were freshly collected from outbreak-affected areas of the Great Barrier Reef by Cairns Marine every few months and housed at either: (1) the Australian Institute of Marine Science (AIMS, Townsville, Queensland); or (2) Underwater World Sea Life Aquarium (Mooloolaba, Queensland). Both groups of animals were kept in protein-skimmed, flow-through tanks on an altered diet (e.g. dried seaweed, scallop meat, fish pellets, shrimp). Tube feet and terminal sensory tentacles were individually removed using scissors and immediately 1) placed in RNAlater or frozen on dry ice for RNA isolation and protein extractions, respectively or 2) placed into 4% paraformaldehyde for microscopic analysis.

### Transcriptome sequencing, analysis and characterisation of putative olfactory GPCR sequences

Total RNA was extracted from COTS tube feet and sensory tentacles using TriZol reagent (Life Technologies) following manufacturer’s instructions*.* Library preparation and sequencing was performed by BGI, Hong Kong on the Illumina HiSeq 2000 sequencing platform. After *de novo* transcriptome assembly using the CLC genomics workbench v.7.0, transcript open reading frames (ORFs) were determined using the online ORF Predictor (http://proteomics.ysu.edu/tools/OrfPredictor.html). A full list of over 700 rhodopsin-like GPCRs (pfam: 7tm_1) from the COTS genome was subjected to a BLASTp against the tube foot and sensory tentacle transcriptomes. Matches with >90% identity and with an e-value of 0 were further investigated. Expression analysis was performed using R software V3.1.1 (https://www.r-project.org/) and specificity to the sensory tissues was calculated by Z-score using the Scale function. Hidden Markov Model (HMM)-based topology predictor TMHMM Server Version 2.0 (http://www.cbs.dtu.dk/services/TMHMM/), was used to analyse tube foot transcriptome sequences for transmembrane helices with default parameters and sequences were analysed for Pfam matches using the EMBL-EBI Pfam 30.0 database (http://pfam.xfam.org/). Any sequences which did not belong to Pfam family 7tm_1 (PF00001), had less than six, or more than seven transmembrane domains were discounted from further analysis. All remaining genes were subsequently named *Acanthaster planci* putative Olfactory Receptors (hereafter referred to as *ApOR*s). Molecular weight was calculated using the online tool from the sequence manipulation suite (http://www.bioinformatics.org/sms/prot_mw.html). BLASTx homology searches of the GenBank non-redundant database at the National Centre for Biotechnology Information (NCBI - http://blast.ncbi.nlm.nih.gov/Blast.cgi) were performed on transcripts. A tBLASTn of candidate GPCRs against COTS genome scaffolds obtained from the Okinawan Institute of Science and Technology (OIST) COTS genome browser (http://marinegenomics.oist.jp/gallery/) was performed to determine clustering of receptor sequences within the genome, with four or more genes found in a tandem array determined to be a cluster. G-protein coupling for putative *ApOR*s was predicted using the online tool PRED_COUPLE 2.00 (http://athina.biol.uoa.gr/bioinformatics/PRED-COUPLE2/). MikTex TexShade software was used to generate schematics showing amino acid conservation for figures. Transcriptome data for other COTS tissues was obtained from Hall et al. [38]. Relative expression heatmaps were constructed using R (V3.1.1) (https://www.r-project.org/). For phylogenetic analysis, a subset of 10 ORs or CRs each from *H. sapiens*, *Mus musculus*, *C. elegans*, and *Aplysia californica* were obtained from the NCBI protein database. A subset of 40 *surreal* GPCRs from *S. purpuratus* (10 each from groups A, B, C and D) were obtained from the online Echinoderm genomic database, Echinobase (http://www.echinobase.org/Echinobase/). *ApOR*s*, surreal* GPCRs and *ORs/CRs* from other species sequences were trimmed to the transmembrane region, including loops. Multiple sequence alignments were performed with the Muscle algorithm in the software Molecular Evolutionary Genetic Analysis (MEGA) version 7 and a tree was produced using the maximum likelihood method with 1000 bootstrap replicates.

### Anatomical and microscopic analysis of tube feet and terminal sensory tentacles

The general anatomical photographs of COTS sensory tissues were performed using a Leica dissection microscope M205A. For histological analysis, samples that had been fixed in 4% paraformaldehyde overnight were transferred into 70% ethanol for longer-term storage. These were then further dehydrated in ethanol dilution series before being embedded in paraffin wax. The embedded samples were sectioned (10 μm sections with transverse cross segments made using a rotary microtome and stained with Harris hematoxylin and eosin) using standard procedures as previously described [33]. Slides were mounted using DePex (BDH Chemicals) and sections were viewed and photographed with a light microscope (BX51; Olympus) equipped with a camera system (UC50; Olympus).

For scanning electron microscopy (SEM), samples that had been fixed in 4% paraformaldehyde overnight were further fixed in fixation buffer (glutaraldehyde and paraformaldehyde) for 4 h at room temperature. Samples were then washed in a primary wash (0.1 Millonig buffer) three times for 10 min. Secondary fixation was applied by immersion in Osmium tetroxide buffer for 1 h, then washed in 0.1M Millonig buffer, and one last time in MilliQ water. Samples were dehydrated in ethanol from 50% to 100%, and then dried with a critical point dryer in 100% ethanol. Finally, samples were mounted onto stubs with carbon tape for thin layer gold sputter coating (Hitachi ion sputtering apparatus, E5000) for 1min. The specimens were examined by a Hitachi S-2500 SEM at 15 kV.

### Probe preparation and in situ hybridisation (ISH)

Total RNA was extracted from COTS tube feet using TriZol reagent (Life Technologies) following manufacturer’s instructions. Following extraction, RNA was assessed for quality by visualisation on a 1.2% agarose gel, and quantified using a Nanodrop spectrophotometer (Thermo Scientific). First-strand cDNA was synthesised from 1 μg total RNA using random hexamers and the TaqMan Reverse Transcription Kit (Applied Biosystems). Gene-specific primers were designed from the transcriptome-derived nucleotide sequences using CLC Genomics Workbench software and PCR was carried out following a routine protocol optimized for individual genes (Additional file 1: Table S3). PCR products were analyzed by agarose gel electrophoresis and amplicons purified using the QIAquick Gel Purification kit (Qiagen). PCR products were cloned into a pGEM-T Easy vector (Promega) according to manufacturer’s instructions, followed by colony PCR using T7 and SP6 primers (Promega) and plasmid purification using the QIAprep Spin Miniprep kit (Qiagen). Purified plasmid was sent to the Australian Genomic Research Facility (AGRF, UQ, Brisbane) for sequencing and determination of orientation. Purified plasmid was amplified using M13 primers before gel purification of bands in correct size range using the QIAquick Gel Purification kit (Qiagen).

Sense and antisense digoxigenin (DIG)-labelled riboprobes were prepared using a DIG RNA labelling mix kit (Roche) as per protocol [39], using SP6 and T7 polymerase (SP6 5’-TAATACGACTCACTATAGGG -3', T7 5'-ATTTAGGTGACACTATAG3'). COTS tube foot and sensory tentacles were fixed in 4% paraformaldehyde overnight at 4°C, then placed in 30% sucrose in PBS overnight before being embedded in OCT compound and frozen at -80°C. Serial transverse sections of the tissues were cut at 10 μm thickness using a cryostat sectioner. Sections were then incubated for one hour at room temperature (~24°C) before being washed in PBS with 0.1% Tween 20 (PBST). Sections were pre-hybridised for 3 h in prehybridisation solution [50% formamide, 5x sodium saline citrate, 5 mM EDTA, 1% Denhardt’s solution (Sigma), 100 μg/ml heparin, 100 μg/ml tRNA, 0.1% Tween20] at 55°C. Hybridisation was performed using the same solution with 200 ng/ml DIG-labelled riboprobe added and incubated overnight at 42°C. Washing, detection and mounting for viewing was performed as described by Cummins et al. [39]. Sections were viewed under a confocal laser-scanning microscope (Nikon).

### Identification and phylogenetic analysis of G proteins

Echinoderm G protein sequences were curated from the NCBI protein database. COTS sequences were obtained by a keyword search (G alpha) within the COTS gene annotations. Subfamilies and expansions within the terrestrial and aquatic vertebrates (*H. sapiens*, *M. musculus, Danio rerio* and *Takifugu rubripes*) and terrestrial and aquatic invertebrates (*Crassostrea gigas, A. californica, Saccoglossus kowalevskii* and *Patiria miniata*) were obtained from the NCBI protein database. Protein sequences were aligned in MEGA v.7 using the Muscle algorithm and tree constructed using the maximum-likelihood method with 1000 bootstrap replicates. The final 25 amino acids for each sequence in multiple different species were aligned and visualised using MikTex Texshade software.

### Western blot and immunofluorescence

For Western blotting, frozen COTS tube feet were thawed in lysis buffer (2% SDS in 50 mL 1xPBS + 500 μl of β mercaptoethanol), homogenised and centrifuged at 12,000 xg for 3 min. Supernatant was collected and total protein concentration was measured at A_280nm_. Approximately 50 μg of total protein and a molecular weight marker (Bio-Rad) was loaded into an ECL Gel 4-20% (GE Healthcare Life Science) and separated at 150V. Protein was then transferred onto nitrocellulose (0.2 μm; Bio-Rad). The membrane was blocked in 4% blocking solution (skim milk powder in PBT) at room temperature for 1h. Primary antibody for rabbit anti-Gαi (Santa Cruz) was incubated with the membrane (1:1000 in PBT) at 4°C overnight. Following washes in PBT, a secondary antibody (1:15,000; anti-rabbit Ig-IR 680) was added and incubated at room temperature for 1 h. Following washes in PBT, antibody binding was detected using an Odyssey CLx, LI-COR.

For immunofluorescence, tube feet and sensory tentacles fixed in 4% paraformaldehyde overnight at 4°C, were dehydration in ascending concentrations of ethyl alcohol for 30 min each, cleared in xylene three times, infiltrated, and embedded in paraffin. Serial transverse sections of the tissues were cut at 10 μm thickness using a microtome. Sections were then deparaffinized in xylene and rehydrated in a descending concentration of ethanol. Subsequently, immunodetection was performed using methods described in Adamson et al. 2016 [40]. The primary antibody was rabbit anti-G**α**i (Santa Cruz) at 1:500 dilution, and the secondary antibody was goat anti-rabbit Alexa 488 (Santa Cruz) at 1:200. DAPI was used as a nuclear stain. In negative controls, tissues were processed by the same protocol, using secondary antibody only (no primary antibody). Images were acquired using a Nikon A1+ confocal microscope and DS-Fi2 camera.

## Results

### Identification of COTS putative olfactory GPCRs

**Fig. 1:**
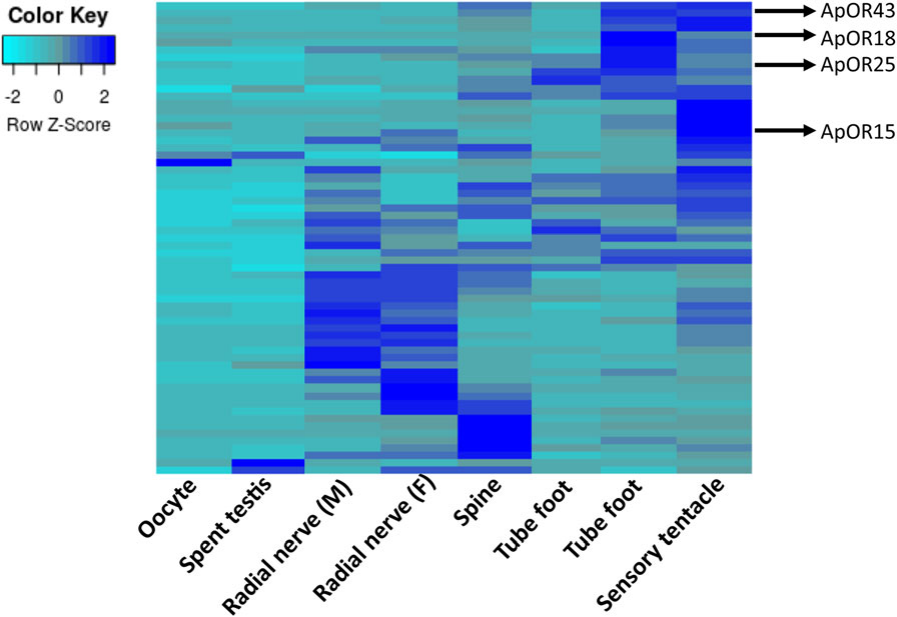
Heat map showing relative tissue distribution of candidate olfactory GPCRs. Candidate olfactory GPCRs used for subsequent in situ hybridisation are indicated

Transcriptomes for the COTS tube foot and terminal sensory tentacle were prepared and summarised in Table 1. A total of 775 rhodopsin-like GPCRs had been identified in the COTS genome [38]. These were subjected to BLASTp against our transcriptomes showing that 77 had hits with an e-value of 0 and ≥90% identity (Additional file 2: File S1). Of these, 26 were found in both tissue transcriptomes, 42 matched only to the sensory tentacle, and 9 matched only to the tube foot. Those genes which had six or seven transmembrane domains were subsequently named putative *Acanthaster planci* olfactory receptors (*ApORs*; *1-63*). Expression values showed that 20 *ApORs* were exclusive to these two sensory organs (Z-score cutoff = 1.69): seven were specific to the tube foot and 13 specific to the sensory tentacles (Fig. 1). Nerve transcriptome of male and female also showed abundant expression of putative *ApORs*, some of which overlap in expression with the tube foot and sensory tentacles.

**Table 1:** Transcriptome summaries for tube foot and sensory tentacles

	Tube foot	Sensory tentacle
N50 (bp)	444	388
Raw reads (paired end)	29689077	32755432
Assembled sequences	369341	427792
Maximum length (nt)	14660	16569
Minimum length (nt)	45	45
Mean length (nt)	238.06	231.78
Standard deviation length (nt)	472.51	459.00

**Fig. 2:**
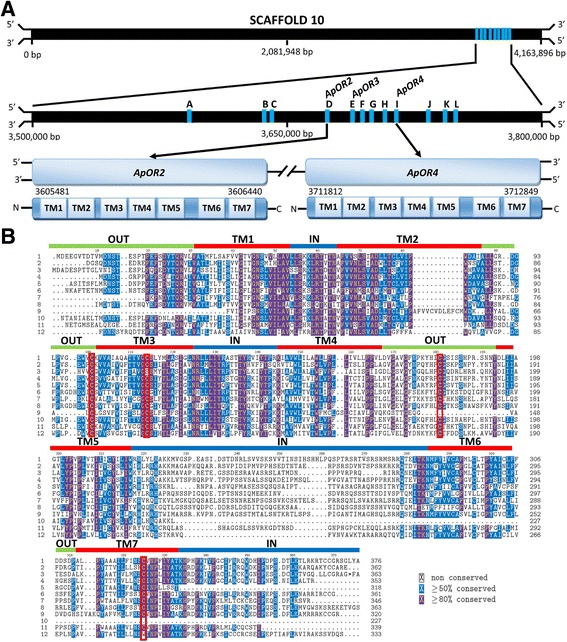
Characterisation of candidate olfactory GPCRs within COTS genome scaffold 10. **a** 12 genes (A-L, including ApOR2, 3 and 4) encoding 7TM proteins are clustered and found in both transcriptional orientations. **b** Comparative multiple sequence alignment of scaffold 10 olfactory GPCRs. Green bars indicate extracellular loops (OUT), *blue bars* indicate intracellular loops (IN) and *red bars* indicated TM domain regions

The average size for the 63 *ApOR*s was 522 amino acids with highest matches to receptors including alpha 1A adrenergic receptor of *S. purpuratus* (Additional file 3: Table S1). Analysis of organisation of the 63 *ApOR*s within the COTS genome showed that many are present as tandem arrays. For example, within the 4,163,896 bp of scaffold 10, 12 rhodopsin-like GPCRs (A – L, including *ApORs 2, 3* and *4*) are found clustered over a 140,332 bp region (Fig. 2a). Multiple sequence alignment of the derived proteins showed that all putative transmembrane helices are highly conserved (≥80%) (Fig. 2b). The predicted first and second intracellular loops also display conserved elements, however divergence is evident in the intracellular C-terminus and the third intracellular loop displays almost no similarity between sequences. The extracellular regions, particularly the second, third and fourth loops, also show high substitution rates (>50%). Four cysteine residues with 80% conservation across sequences are found in the second and third extracellular loops and transmembrane domains three and seven, respectively (Fig. 2b). Another three cysteine residues found in transmembrane domains two, five and six, have lower conservation across sequences (≥50%). Most of the 63 putative *ApOR*s were predicted to couple with Gαi/o proteins (Additional file 3: Table S1).

**Fig. 3:**
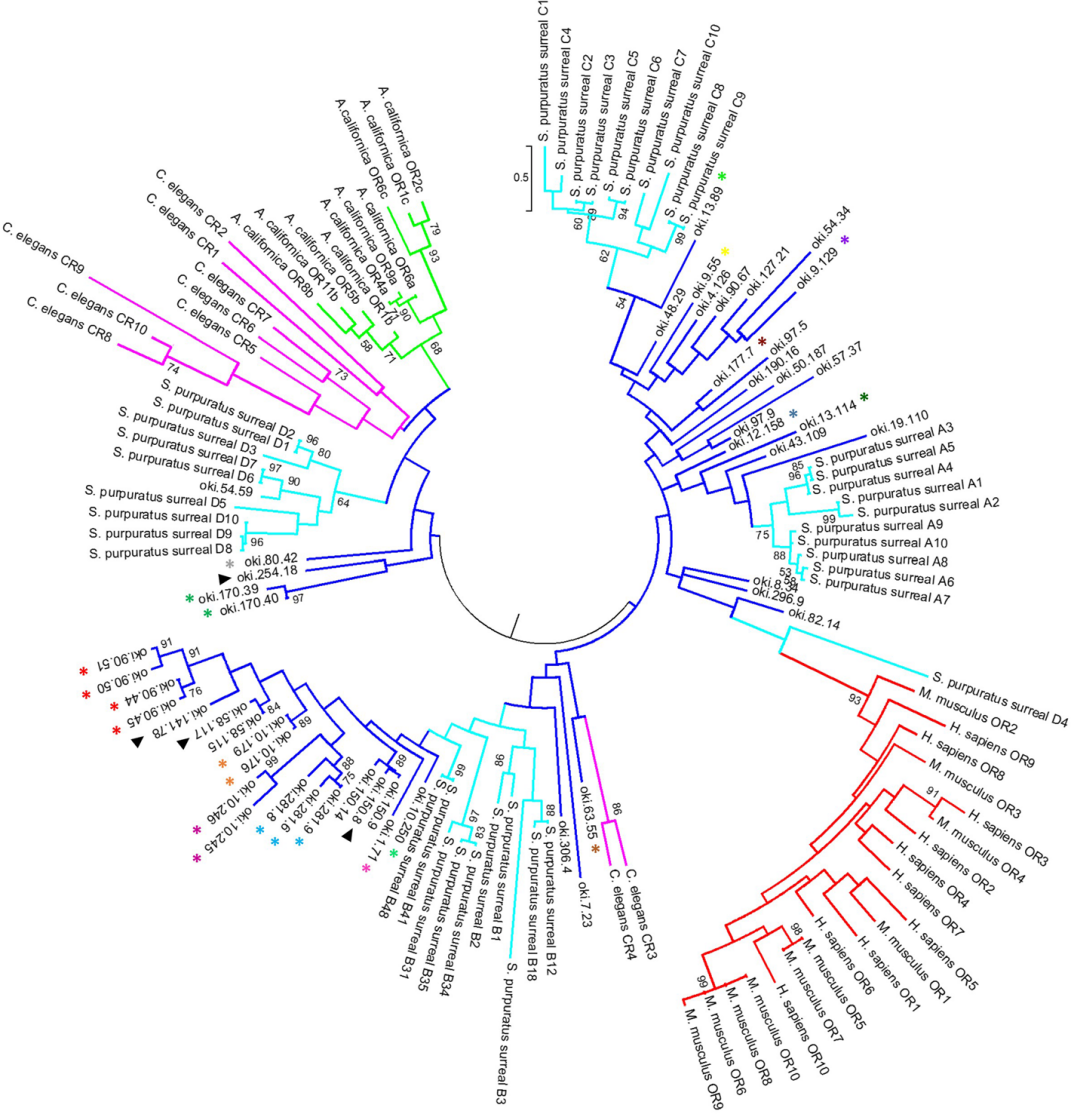
Phylogenetic analysis of putative *ApOR* sequences, sea urchin *surreal*-GPCRs, and *OR*s and *CR*s from several other vertebrate and invertebrate species. *ApOR*s are indicated by dark blue lines, *surreal*-GPCRs are indicated by light blue, *C. elegans CR*s are indicated by purple, *A. californica CR*s are indicated by green, and *H. sapiens* and *M. musculus OR*s are indicated by red. *ApOR*s which cluster in the COTS genome are indicated by coloured asterisks, with each different colour representing a different genomic cluster. *ApOR*s used for in situ hybridisation are indicated by black arrows. Scale bar represents number of amino acid substitutions per site

Phylogenetic analysis of putative *ApOR*s with *OR*s from other species shows that they share the most similarity with the *S. purpuratus surreal*-GPCRs. Each subfamily of *ApOR*s clusters next to a corresponding subfamily of *surreals,* however there is also significant divergence between each clade (Fig. 3), however several sequences are more divergent and do not cluster into the main groups. Genes that are found as tandem arrays in the genome show the most similarity to each other within these groups and cluster together. *A. californica* and *C. elegans CR*s show the most similarity to each other and cluster separately from the other organisms, with the exception of *C. elegans CR*3 and 4, which are grouped with several of the more divergent *ApOR*s. *OR*s from human and mouse cluster together separately from all invertebrate receptors, however one *surreal*-GPCR, D4, is grouped near the vertebrate sequences.

### Anatomical and microscopic analysis of COTS sensory organs

**Fig. 4:**
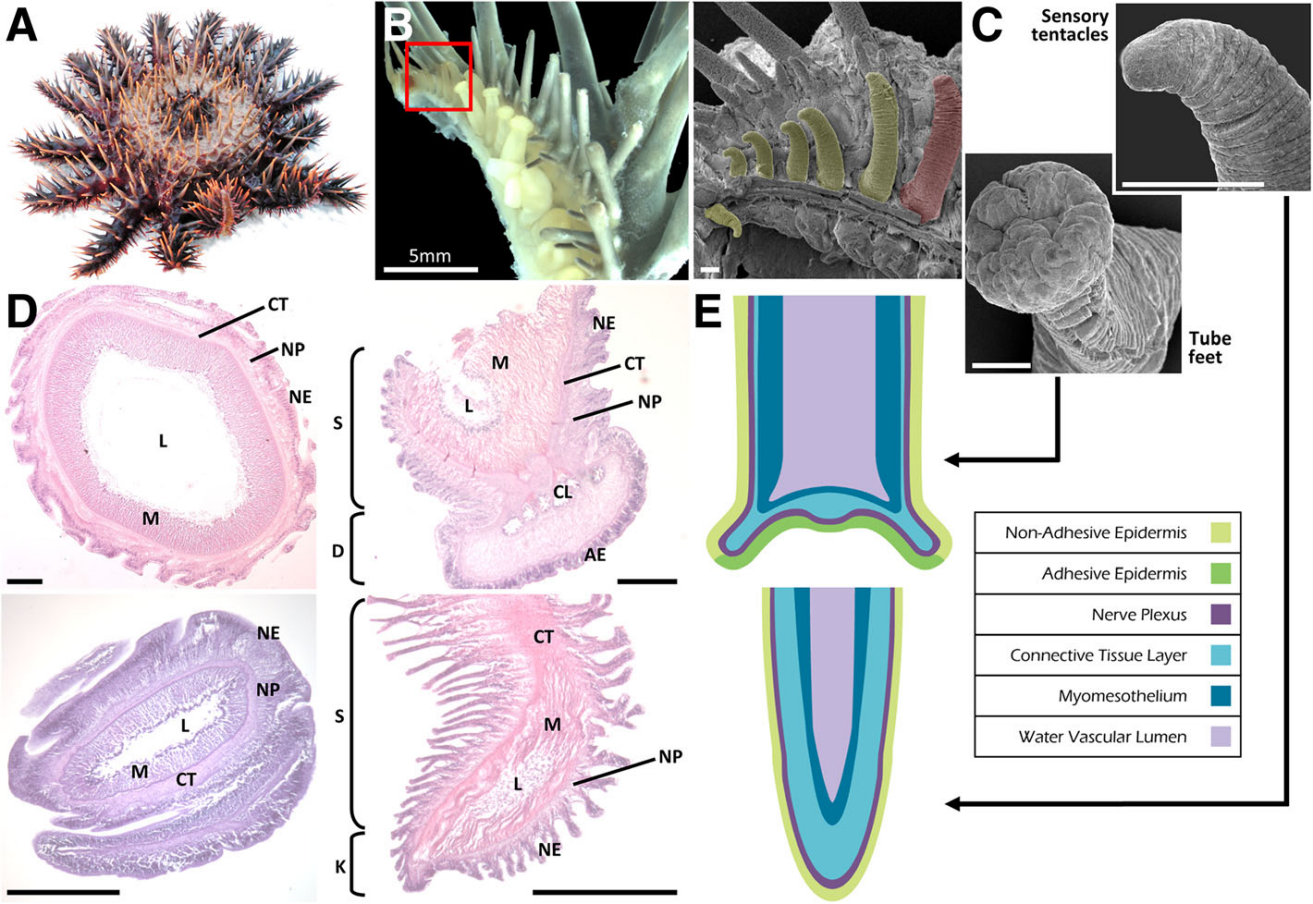
Anatomical and microscopic analysis of COTS sensory organs. **a** COTS showing one arm lifted to reveal tube feet and sensory tentacles. **b** *Left*: Light microscope image of half a COTS arm tip showing region of tube feet and sensory tentacles. *Right*: SEM image of boxed area showing sensory tentacles (*yellow*) and tube feet (*red*). **c** SEM imaging of COTS tube foot and a sensory tentacle. **d** Histological staining (H&E) and (**e**) schematics showing characteristic tissue layers of COTS tube foot and sensory tentacle organs. *AE* adhesive epidermis; *NE* Non-adhesive epidermis; *CL* connective tissue radial laminae; *CT* connective tissue layer; *D* disc; *S* stem; *L* water-vascular lumen; *M* myomesothelium; *NP* nerve plexus; *C* cuticle; *E* epidermis. Scale bars = 200 μm unless otherwise marked

COTS have reinforced disc-ending tube feet that extend in two rows along the ambulacral column on the underside of each of their arms. Tube feet are typically larger in the part of the arm that is close to the central disc and are smaller towards the tip of the arm. The radial nerve rests between the two rows, directly underneath the ambulacral ossicles, terminating in an eye spot (ocelli) which is surrounded by spines and sensory tentacles at the very tip of the arm (Fig. 4a, b). SEM investigations revealed that the tube feet gradually decrease in size along the length of the entire arm; the final six to eight of the tube feet, at the distal ~2mm of the arm, have knob-ending morphology and no adhesive disc, characteristic of sensory tentacles (Fig. 4c). Depending on the size of the animal, tube feet can be up to >1 cm and sensory tentacles can be 500 -1,500μm when not extended. No cilia-like projections were visible on either of these tissues using an SEM approach.

Histology using Harris’s haematoxylin and eosin revealed in more detail the structure of the cell layers within both sensory tissues (Fig. 4d). COTS tube feet and sensory tentacles consist of an epithelia, a basiepidermal nerve plexus, connective tissue layers, and finally a myomesothelium which surrounds the water vascular lumen. These tissue layers were graphically represented in schematic diagrams using colour to distinguish clearly between the tissue layers (Fig. 4e). Longitudinal sections showed that tube feet possess the characteristic disc-shaped adhesive epidermis lacking in the sensory tentacles, however all other tissue layers appear the same in both tissues. No cilia were visible on either tissue.

### Spatial expression of ApORs expression in olfactory organs

**Fig. 5:**
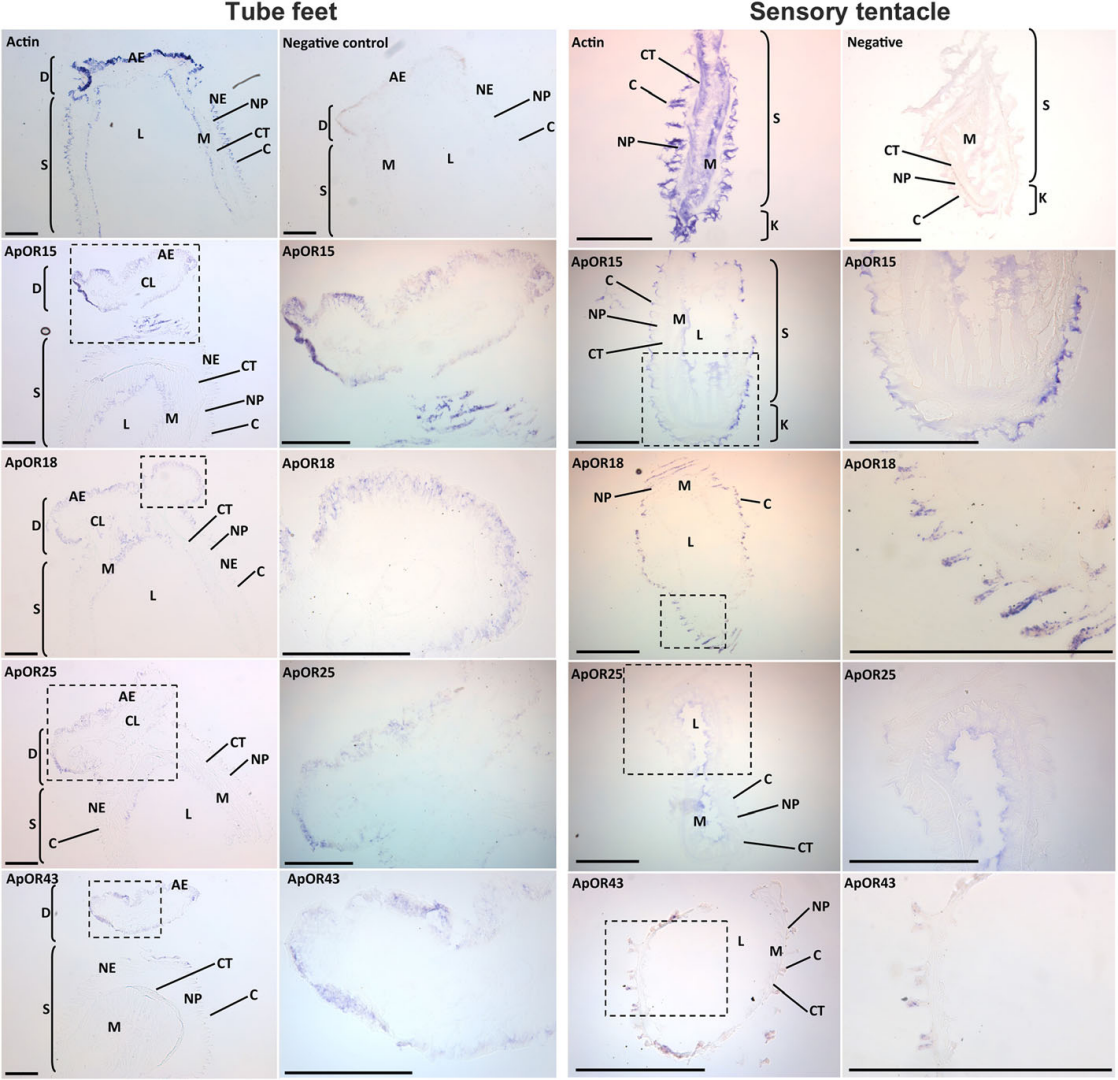
In situ hybridisation of COTS tube feet and sensory tentacles using digoxigenin-labelled antisense RNA probes to show spatial expression of candidate olfactory receptors, ApOR 15, 18, 25, 43, and positive control actin. AE, adhesive epidermis; NE, Non-adhesive epidermis; CL, connective tissue radial laminae; CT, connective tissue layer; D, disc; S, stem; L, water-vascular lumen; M, myomesothelium; NP, nerve plexus; C, cuticle; E, epidermis. Scale bars = 200 μm

To investigate the spatial distribution of *ApOR* genes within regions of the olfactory organ sensory epithelia, in situ hybridisation (ISH) was performed. Based on transcriptome analysis showing specificity to tube foot and sensory tentacle tissues (see Fig. 1), *ApOR15, ApOR18, ApOR25 and ApOR43* genes were chosen for further investigation. These particular genes do not cluster within the COTS genome, however their specificity to these tissues warranted further investigation. *ApOR15, 18, 25, and 43* are expressed in the adhesive epidermis of the tube feet with some expression in the nerve plexus and non-adhesive epidermis of the stem closest to the disc (Fig. 5). Surprisingly, these genes are also expressed within the inner epithelia, the myomesothelium, which is in direct contact with the water vascular lumen. *ApOR43* shows relatively low expression level in the myomesothelium and non-adhesive epidermis. *ApOR18 and ApOR25* are expressed in both the adhesive epidermis and myomesothelium of the tube foot. *ApOR15* shows the strongest expression, particularly within the adhesive epidermis and the distal portion of the stem. Positive control actin ISH showed consistent expression throughout the tube foot. Negative control using a sense DIG-labelled riboprobe showed no specific staining.

*ApOR43* is predominantly expressed in a few cells within the cuticle of the sensory tentacles (Fig. 5). Expression of *ApOR15* and *ApOR25* are highly expressed in the cuticle of the outer epithelia, as well as the myomesothelium. *ApOR18* expression is strong within just a few cells of the epidermis of the sensory tentacles. Positive control actin ISH showed consistent expression throughout all regions of the sensory tentacle. Negative control using a sense DIG-labelled riboprobe showed no specific staining.

### G protein identification and spatial expression in COTS sensory organs

**Fig. 6:**
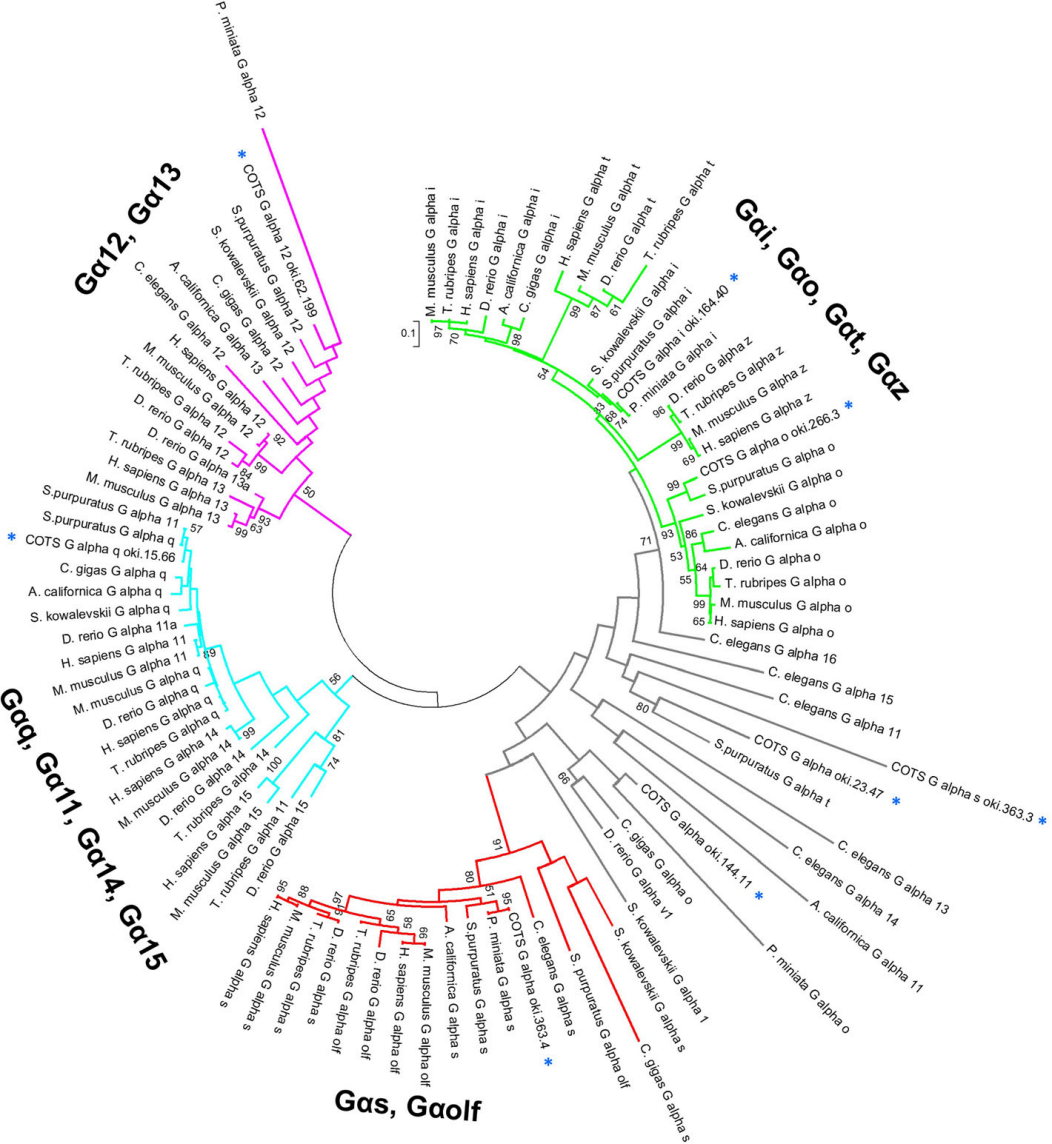
Phylogenetic tree of G alpha subunit proteins from COTS and various species. Main families of G alpha proteins are indicated by coloured lines: Gα12/13 indicated by purple lines, Gαi/o/t/z by green, Gαs/olf by red and Gαq/11/14/15 by light blue. Grey lines indicate sequences which do not cluster into these main families. Scale bar represents number of amino acid substitutions per site

Multiple sequence alignment of G proteins shows high overall sequence similarity (Additional file 4: Fig. S2), however aquatic species (i.e. *COTS, Strongylocentrotus purpuratus, Patiria pectinifera, A. californica*) showed distinct high conservation with terrestrial species in the C-terminal region. This is a common region from which G protein-specific antibodies have been generated. Phylogenetic analysis demonstrates that COTS possess orthologs for each of the four main families of G alpha subunit proteins, but do not possess orthologs for each of the subfamilies (Fig. 6). For example, COTS have representatives for Gα12, Gαq, Gαs, Gαi and Gαo. However, they are lacking clear representatives for Gαolf, Gαt, Gαz, Gα11/14/15 and Gα13. In contrast, the three remaining COTS G alpha subunit proteins which were included in the analysis show significantly lower similarity to any of the four main families and cluster separately, along with several invertebrate G alpha subunit proteins including several of those from *C. elegans*. Gαq and Gαi show high levels of conservation, particularly between invertebrates which cluster separately from the vertebrate equivalents. Gα12 and Gαs show higher substitution rates, however invertebrates and vertebrates remain in separate clusters. Analysis of the sensory organ transcriptomes revealed the presence of all Gα protein transcripts in the sensory organs, as well as other tissues; Gαq and Gαi showed higher expression within the sensory organs (Additional file 5: Table S2).

**Fig. 7:**
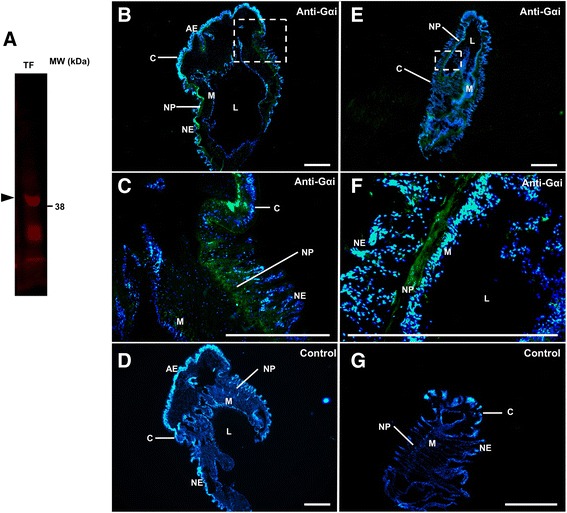
**a** Western blot showing staining of a protein band at approximately 40 kDa (*arrow*) in tube feet extracts using anti-Gαi. **b** Immunofluoresence (*green*) staining of Gαi protein in COTS tube foot tissue section. Blue represents DAPI nuclear fluorescence. **c** A higher-resolution micrograph of the area boxed in (**b**). **d** Negative control showing only nuclear staining. **e** Immunofluoresence (*green*) staining of Gαi protein in COTS sensory tentacles. **f** A higher-resolution micrograph of the area boxed in (**e**). **g** Negative control showing only nuclear staining. AE, adhesive epidermis; NE, Non-adhesive epidermis; CT, connective tissue layer; M, myomesothelium; NP, nerve plexus; C, cuticle. Scale bars = 200 μm

To investigate the spatial expression of Gα proteins within the COTS tube feet, a commercial antibody directed to the C-terminus of Gαi protein was tested in Western blot. Western blot analysis confirmed high specificity for a tube foot protein with Gαi, consistent with the predicted size of COTS Gαi protein (~40.1 kDa; Fig. 7a). This was subsequently used in immunofluorescence localisation within COTS tube feet (Fig. 7b-d) and sensory tentacles (Fig. 7e-g). Anti-Gαi showed immunoreactivity within the sensory epithelium and nerve plexus of both tissues. Negative controls, in which secondary antibody only was used, showed no specific staining.

## Discussion

In this study, our primary objectives were to investigate the structure and morphology of the COTS olfactory organs, identify putative olfactory rhodopsin-like GPCRs within the transcriptomes of the COTS olfactory organs and then to elucidate their spatial expression those tissues. We also aimed to support the functional role of these receptors as GPCRs through analysis of sensory tissue G proteins.

Based on predictions from the COTS genome [38], the proportion of GPCRs in relation to genome size is comparable to other metazoans, including humans [20]. Rhodopsin-type GPCR expansions are common throughout the animal kingdom, with corresponding evolutionary modifications and structural adaptations leading to a diversity of functions [21]. As olfactory receptors have evolved directly and independently from ancestral GPCR sequences multiple times across many animal lineages, it is clear that having 7TM domains is a key attribute that allows for efficient ligand binding and cellular activation; therefore, olfactory receptors may develop from any GPCR sequence within a genome [41]. The cluster of 16 putative *ApOR*s found on COTS genome scaffold 10 likely arose via rapid duplication from an ancestral GPCR sequence, which may or may not have had a prior olfactory chemosensory function. This observation of clustered olfactory gene families is consistent with those found within the genomes of *H. sapiens* [42], the marine mollusc *A. californica* [4] and the aquatic crustacean *Daphnia pulex* [43].

Chemoreceptor gene families have evolved independently numerous times across many different phyla, probably due to species-specific physiology and behaviour, with duplicate retention linked to cellular diversity and increasing organismal complexity [2]. For example, a recent study of GPCRs in the demosponge, *Amphemidon queenslandica*, showed large species-specific expansions in the rhodopsin-like receptor family driven by gene duplication [6]. It was proposed that *A. queenslandica* rhodopsins, including the olfactory type, had diverged considerably due to sponge-specific physiology [6]. Many species-specific expansions of olfactory GPCRs, such as those found in the sea urchin *S. purpuratus*, sea sponge *A. queenslandica* and the sea slug *A. californica* lack clearly identifiable orthologs in other metazoans [4, 6, 21].

Relative high levels of conservation within TM domains and intracellular regions is common among olfactory GPCRs within a species, particularly as the TM domains are the structural core of the protein and interaction with the associated intracellular proteins (such as G proteins) occurs through the intracellular loops [44]. Our analysis of *ApOR*s reflects this, and higher substitution rates are evident in the extracellular regions, particularly within extracellular loops one and four of those receptors analysed. Extracellular loops potentially form the ligand-binding site of a GPCR, and sequence modifications within these regions enable binding to novel molecules, with positive selection acting to retain the beneficial duplicated sequences within the functional repertoire of the species. This process has been recorded in many other animal groups, including mammals such as primates [45] and the nematode *C. elegans* [19]. In contrast, sea urchin rhodopsins showed high divergence across all TM domains and several of the intracellular loops but this variation was not shown by all subfamilies across the same sites [21]. Ancestral GPCR orthologs are often lost during the process of gene expansions, however *S. purpuratus* was shown to retain some of this ancestral genomic complexity, which is secondarily reduced in other phyla [21]. Many fish families similarly show large repertoires of ancestral chemosensory GPCR sequences [46]. The subfamilies of COTS rhodopsin-type GPCRs, as evidenced by phylogenetic analysis, may indicate that this species has also retained its ancestral GPCR sequences.

While COTS putative olfactory receptors show little similarity to those previously described in other bilaterians, they also display relatively little similarity to sea urchin olfactory receptors when subjected to a BLAST search. Despite this, our phylogenetic analysis of *ApOR*s with those from other species demonstrates that *ApOR*s are most similar to *surreal*-GPCRs when compared to the other groups. The *S. purpuratus* rhodopsin-like receptors were shown to contain many largely expanded subfamilies specific to the Echinoid class of the echinoderm phylum [21]. Based on our genomic and transcriptomic analyses, it appears that COTS also contain rapidly expanded lineages of putative olfactory rhodopsin-like GPCRs, many of which may be unique to the class Asteroidea. This is consistent with previous studies in which cross-species comparisons have shown large variation in size and functionality of olfactory GPCR families by the combination of duplications, deletions and mutations known collectively as birth-and-death evolution [2]. Our results may indicate that COTS rhodopsin-like GPCRs have rapidly and independently evolved not only since the divergence of echinoderms from other dueterostomes but also more recently in evolutionary history when Asteroids and Echinoids diverged from their common ancestor in the early Palaeozoic. GPCR families in many species appear to be subject to weak positive selection, including those found in aquatic animals such as fish and invertebrates including nematodes [2]. However, in a recent study by Yoder [45], an expanded subfamily of olfactory GPCRs were found to be under strong positive selection, particularly within transmembrane regions, in a group of nocturnal strepsirrhine primates, the mouse lemurs. This may also be the case for *ApOR*s, whose function is critical to the COTS life cycle.

The expression of those *ApORs* selected for this study supports our hypothesis that this family could have an olfactory role. First, the relative abundance of transcript within the tube feet suggested a selective requirement within this organ, and secondly their spatial expression was localised within sensory epithelia. However, we find that expression was not exclusive to the sensory epithelia, but also present within the tube foot and sensory tentacle myomesothelium. This may contradict previous histological findings and raises several questions pertaining to the true nature of cells within these tissues. According to prior studies, both sensory and secretory cells are more abundant within the adhesive epidermis of the tube foot [32], and the sensory cells are assumed to be chemo- or mechano-sensory [33]. According to our results, sensory neurons may also be present in the myomesothelium. This layer is known to have ciliated adluminal cells whose function has not yet been fully characterised in echinoderms [47]. If *ApOR*s are present in this region, they may be used to sense and bind ligands that have been transported into the water vascular lumen of the tube feet and sensory tentacles via the madreporite and through the remainder of the water vascular system. If this is the case in COTS, it may also be true for other echinoderms.

The findings of transcriptomic analyses also suggest that COTS utilise all four of the main families of G proteins for intracellular signal transduction, as well as three uncharacterised G alpha protein sequences which do not appear to belong to these main families. These Gα proteins are directly involved in signal transduction from GPCRs and in turn activate the G protein βγ complex which acts as a secondary messenger; a process which has been well established in many other species [5]. Most Gα protein sequences from invertebrates, particularly the echinoderms (*A. planci*, *S. purpuratus* and *P. miniata*) show slightly higher divergence than those found in other species and cluster together, which may reflect the difference between olfaction in vertebrates as opposed to invertebrates. Most of the identified *ApOR* genes are predicted to couple with Gαi/o proteins, a result which supports the presumption that G proteins are used in signal transduction from GPCRs in the sensory epithelia of COTS olfactory organs. Gαi appears to be more conserved amongst the echinoderm species and less conserved amongst the other species. In contrast, Gαo shows more variation amongst invertebrates and less in vertebrate species. However, we only show spatial localisation of tube foot and sensory tentacle Gαi in this study as the commercially-available antibody was directed to the conserved region in COTS. The clustering of three uncharacterised G alpha proteins from COTS separate from the four main subfamilies, along with those expansions from *C. elegans*, may indicate that these are species-specific genes. Some of these also show strong expression within the sensory tissues of COTS, which further supports their putative role in chemosensory signalling in this species. Invertebrate representatives of Gαs/olf are grouped in a separate clade to that of vertebrates, indicating these genes may have only diverged into true subfamilies more recently in the vertebrate lineages. The same may also be true of Gαi/t and Gα11/q; while it is more difficult to distinguish within the phylogeny, it may be the case that COTS and other invertebrates have ancestral forms of these genes.

Olfactory receptors, particularly pheromone receptors, have often been considered of key importance in creating and maintaining species boundaries among mammals as they act as prezygotic barriers resulting in reproductive isolation [45]. For example, the platypus (*Ornithorhynchus anatinus*), a semi-aquatic monotreme mammal, has 1400 copies of V1R genes alone, a greater number than those found in mouse or dog [48, 49]. Chemosensory receptor gene families are thought to undergo dynamic changes during evolution and repertoire size also changes in response to an organism’s environment; for example, it has been documented that animals possessing well-developed vision will have significantly reduced functional repertoires of chemoreceptor genes [46]. In the case of the dolphin, their well-developed eyesight and hearing (echolocation system), may have rendered olfaction redundant [46, 49]. However, studies of terrestrial invertebrates such as insects indicate that olfaction is equally, if not more important in invertebrates than it is in mammals [23]. COTS lack an acoustic sense and have limited vision, thus chemoreception is likely integral to all aspects of their life cycle. The findings of our study support a growing body of evidence that olfactory receptors act as critical prezygotic barriers causing reproductive isolation in free-spawning marine invertebrates such as COTS. Therefore, interference in their chemically mediated behaviours, such as spawning, has significant potential for biological control.

## Conclusions

This is a novel and expanding area of research and the challenge now will be to determine which of these putative olfactory receptors bind molecules critical for reproduction, such as pheromones that are required for aggregation and those that may elicit synchronous spawning. Future research is this area should focus on deorphanising putative olfactory receptors and characterising their function via in vitro bioassays, as well as investigating the differences in receptor expression between male and female COTS, and throughout the reproductive and non-reproductive season, in order to determine which are viable targets for development of biological controls. Also, the finding that *ApOR*s localised to the nerve plexus of tube feet stems and within the myomesothelium indicates that COTS may detect waterborne chemical cues with the inside and the outside of the sensory organs. This challenges previous suggestions that sensory cells were only found in the adhesive epidermis of Asteroid tube feet [32]. These results provide a basis for future studies of olfaction in COTS. As olfaction is critical for many aspects of the COTS life cycle, including species maintenance and reproduction, future research in this area may be the key to developing control technology that could be deployed to mitigate outbreaks of COTS on the Australian Great Barrier Reef, and this may be applied to COTS outbreaks on other reefs globally.

## Additional files

Additional file 1: Table S3. Gene-specific primers for four *ApOR*s and expected amplicon sizes. (DOCX 12 kb)

Additional file 2: **File S1.** FASTA file of *ApOR* protein sequences. (TXT 34 kb)

Additional file 3: Table S1. Characterisation of *ApOR*s, indicating oki and gbr gene IDs, size (aa), molecular weight (kDa), Pfam domain, transmembrane domains, evidence of clustering within the COTS genome (>4 genes in a tandem array within a genome scaffold), and G protein coupling prediction. *ApOR*s which were used for in situ hybridisation are marked with an asterisk. (DOCX 20 kb)

Additional file 4: Figure S2a-c. Multiple sequence alignments of G α proteins from COTS and other species. Aquatic species are indicated by a blue line next to the sequence. The final 25 amino acids, to which commercially available antibodies are directed, are indicated by a red rectangle. (ZIP 21297 kb)

Additional file 5: Table S2. Characterisation and expression profile of G proteins in COTS, including three uncharacterised sequences, indicating oki and gbr gene IDs, size (aa), molecular weight (kDa), Pfam domain, and expression (FPKM) in multiple COTS tissue transcriptomes. (DOCX 13 kb)

## Abbreviations

*ApOR*: *Acanthaster planci* putative Olfactory Receptor
COTS: Crown of thorns starfish (*Acanthaster planci*)
GPCR: G protein-coupled receptors
