# Supplementary material for: Identification of putative olfactory G-protein coupled receptors in Crown-of-Thorns starfish, Acanthaster planci
Source: BMC Genomics. 2017 May 23;18:400. doi: 10.1186/s12864-017-3793-4 (PMC5442662; doi:10.1186/s12864-017-3793-4)
Supplement: Supplementary file 1 — Gene-specific primers for four ApORs and expected amplicon sizes. (DOCX 12 kb) [file 12864_2017_3793_MOESM1_ESM.docx]

| Gene name | Forward primer (5’→3’) | Reverse primer (3’→5’) | Amplicon size (bp) |
| --- | --- | --- | --- |
| *ApOR49* | CCACGGATGTCACTACGA | GCTGCTGACGACTGATTT | 809 |
| *ApOR16* | TGCGACATCCTTAAACTC | GTGTAGATGATGGGACTG | 950 |
| *ApOR28* | GTGGGGTAAAGAATGAGG | GCTCTCAGAATCTCTTGT | 1051 |
| *ApOR20* | CCCGTCCAGAGGATCATAA | GGGTGTTGGGGGAATTTG | 660 |
