# Supplementary material for: Identification of putative olfactory G-protein coupled receptors in Crown-of-Thorns starfish, Acanthaster planci
Source: BMC Genomics. 2017 May 23;18:400. doi: 10.1186/s12864-017-3793-4 (PMC5442662; doi:10.1186/s12864-017-3793-4)
Supplement: Supplementary file 3 — Characterisation of ApORs, indicating oki and gbr gene IDs, size (aa), molecular weight (kDa), Pfam domain, transmembrane domains, evidence of clustering within the COTS genome (>4 genes in a tandem array within a genome scaffold), and G protein coupling prediction. ApORs which were used for in situ hybridisation are marked with an asterisk. (DOCX 20 kb) [file 12864_2017_3793_MOESM3_ESM.docx]

| ***Name*** | **Oki ID** | **Gbr ID** | **Size (aa)** | **MW (kDa)** | **Pfam domain** | **TM domains** | **Cluster (>4)** | **G protein** |
| --- | --- | --- | --- | --- | --- | --- | --- | --- |
| *ApOR1* | oki.1.71 | gbr.2.182 | 356 | 39.41 | 7tm_1 | 7 | Yes | Gi/o |
| *ApOR2* | oki.10.245 | gbr.38.74 | 368 | 40.73 | 7tm_1 | 7 | Yes | Gi/o |
| *ApOR3* | oki.10.246 | gbr.38.73 | 367 | 40.92 | 7tm_1 | 7 | Yes | Gq/11 |
| *ApOR4* | oki.10.250 | gbr.38.69 | 408 | 45.75 | 7tm_1 | 7 | Yes | Gi/o |
| *ApOR5* | oki.10.176 | gbr.34.8 | 390 | 42.84 | 7tm_1 | 7 | Yes | Gi/o |
| *ApOR6* | oki.10.179 | gbr.34.8 | 395 | 43.75 | 7tm_1 | 7 | Yes | Gi/o |
| *ApOR7* | oki.102.6 | gbr.67.49 | 424 | 46.32 | 7tm_1 | 7 | No | Gi/o |
| *ApOR8* | oki.114.17 | gbr.236.22 | 896 | 100.92 | 7tm_1 | 7 | No | Gi/o |
| *ApOR9* | oki.12.158 | gbr.3.29 | 431 | 48.2 | 7tm_1 | 7 | Yes | no match |
| *ApOR10* | oki.127.21 | gbr.253.17 | 370 | 42.12 | 7tm_1 | 7 | No | Gi/o |
| *ApOR11* | oki.13.114 | gbr.43.49 | 476 | 52.18 | 7tm_1 | 7 | Yes | Gi/o |
| *ApOR12* | oki.13.81 | gbr.78.34 | 679 | 73.58 | 7tm_1 | 7 | Yes | Gq/11 |
| *ApOR13* | oki.13.89 | gbr.78.45 | 375 | 41.95 | 7tm_1 | 7 | Yes | Gs |
| *ApOR14* | oki.14.118 | gbr.404.12 | 652 | 72.71 | 7tm_1 | 6 | No | Gi/o |
| *ApOR15* | oki.141.78 | gbr.175.43 | 367 | 40.79 | 7tm_1 | 7 | No | Gq/11 |
| *ApOR16* | oki.15.206 | gbr.25.132 | 784 | 87.39 | 7tm_1 | 7 | Yes | Gi/o |
| *ApOR17* | oki.150.14 | gbr.614.1 | 379 | 41.82 | 7tm_1 | 7 | Yes | Gi/o |
| *ApOR18* | oki.150.8 | gbr.527.8 | 375 | 41.62 | 7tm_1 | 7 | No | Gi/o |
| *ApOR19* | oki.150.9 | gbr.527.9 | 386 | 42.55 | 7tm_1 | 7 | Yes | Gi/o |
| *ApOR20* | oki.170.39 | gbr.610.4 | 370 | 41.27 | 7tm_1 | 7 | Yes | Gi/o |
| *ApOR21* | oki.170.40 | gbr.610.5 | 370 | 40.67 | 7tm_1 | 7 | Yes | Gi/o |
| *ApOR22* | oki.177.7 | gbr.165.21 | 432 | 48.67 | 7tm_1 | 7 | Yes | Gi/o |
| *ApOR23* | oki.19.110 | gbr.72.113 | 424 | 47.6 | 7tm_1 | 7 | No | Gi/o |
| *ApOR24* | oki.190.16 | gbr.242.27 | 575 | 64.55 | 7tm_1 | 7 | No | Gq/11 |
| *ApOR25* | oki.254.18 | gbr.61.57 | 375 | 42.79 | 7tm_1 | 7 | No | Gi/o |
| *ApOR26* | oki.281.6 | gbr.56.122 | 380 | 42.69 | 7tm_1 | 7 | Yes | Gs |
| *ApOR27* | oki.281.8 | gbr.56.120 | 392 | 43.95 | 7tm_1 | 7 | Yes | Gi/o |
| *ApOR28* | oki.281.9 | gbr.56.119 | 379 | 42 | 7tm_1 | 7 | Yes | Gi/o |
| *ApOR29* | oki.29.62 | gbr.19.62 | 474 | 52.17 | 7tm_1 | 7 | No | Gi/o |
| *ApOR30* | oki.296.9 | gbr.336.5 | 419 | 47.05 | 7tm_1 | 7 | No | Gi/o |
| *ApOR31* | oki.3.84 | gbr.11.81 | 607 | 67.07 | 7tm_1 | 7 | Yes | Gq/11 |
| *ApOR32* | oki.306.4 | gbr.452.8 | 451 | 50.26 | 7tm_1 | 7 | No | Gi/o |
| *ApOR33* | oki.4.126 | gbr.187.17 | 379 | 42.62 | 7tm_1 | 7 | No | Gq/11 |
| *ApOR34* | oki.43.109 | gbr.99.39 | 467 | 53.33 | 7tm_1 | 7 | No | Gi/o |
| *ApOR35* | oki.48.29 | gbr.87.59 | 413 | 46.72 | 7tm_1 | 7 | No | Gq/11 |
| *ApOR36* | oki.5.176 | gbr.9.80 | 2608 | 293.2 | 7tm_1 | 7 | No | Gi/o |
| *ApOR37* | oki.50.187 | gbr.58.130 | 449 | 50.16 | 7tm_1 | 7 | No | Gq/11 |
| *ApOR38* | oki.54.34 | gbr.24.1 | 356 | 39.33 | 7tm_1 | 6 | No | Gi/o |
| *ApOR39* | oki.54.59 | gbr.155.24 | 536 | 60.47 | 7tm_1 | 7 | No | Gi/o |
| *ApOR40* | oki.55.35 | gbr.327.18 | 1738 | 192.16 | 7tm_1 | 7 | No | Gi/o |
| *ApOR41* | oki.57.37 | gbr.61.16 | 442 | 49.57 | 7tm_1 | 7 | No | Gi/o |
| *ApOR42* | oki.58.115 | gbr.90.21 | 391 | 43.64 | 7tm_1 | 7 | No | Gi/o |
| *ApOR43* | oki.58.117 | gbr.90.19 | 384 | 42.86 | 7tm_1 | 7 | No | Gi/o |
| *ApOR44* | oki.63.55 | gbr.176.5 | 487 | 54.47 | 7tm_1 | 6 | Yes | Gq/11 |
| *ApOR45* | oki.7.23 | gbr.85.23 | 477 | 48.97 | 7tm_1 | 7 | No | Gq/11 |
| *ApOR46* | oki.7.48 | gbr.186.5 | 674 | 74.93 | 7tm_1 | 9 | No | Gi/o |
| *ApOR47* | oki.70.23 | gbr.359.16 | 1467 | 163.02 | 7tm_1 | 7 | No | Gi/o |
| *ApOR48* | oki.77.62 | gbr.583.6 | 704 | 77.58 | 7tm_1 | 7 | No | Gi/o |
| *ApOR49* | oki.78.48 | gbr.93.21 | 421 | 46.84 | 7tm_1 | 6 | No | Gq/11 |
| *ApOR50* | oki.79.46 | gbr.12.54 | 575 | 64.28 | 7tm_1 | 7 | No | Gq/11 |
| *ApOR51* | oki.8.34 | gbr.247.27 | 393 | 44.42 | 7tm_1 | 7 | No | Gi/o |
| *ApOR52* | oki.80.42 | gbr.98.57 | 417 | 46.01 | 7tm_1 | 7 | Yes | Gq/11 |
| *ApOR53* | oki.80.81 | gbr.98.95 | 598 | 65.79 | 7tm_1 | 7 | Yes | Gi/o |
| *ApOR54* | oki.82.14 | gbr.36.3 | 493 | 56.09 | 7tm_1 | 7 | No | Gi/o |
| *ApOR55* | oki.9.55 | gbr.40.59 | 426 | 47.61 | 7tm_1 | 7 | Yes | Gq/11 |
| *ApOR56* | oki.9.129 | gbr.40.137 | 378 | 42.33 | 7tm_1 | 6 | Yes | Gq/11 |
| *ApOR57* | oki.90.44 | gbr.44.21 | 352 | 39.18 | 7tm_1 | 7 | Yes | Gi/o |
| *ApOR58* | oki.90.45 | gbr.44.22 | 377 | 41.74 | 7tm_1 | 7 | Yes | Gi/o |
| *ApOR59* | oki.90.50 | gbr.44.27 | 349 | 38.8 | 7tm_1 | 7 | Yes | Gi/o |
| *ApOR60* | oki.90.51 | gbr.44.28 | 355 | 39.57 | 7tm_1 | 6 | Yes | Gi/o |
| *ApOR61* | oki.90.67 | gbr.44.46 | 382 | 42.69 | 7tm_1 | 7 | No | Gi/o |
| *ApOR62* | oki.97.5 | gbr.140.67 | 473 | 52.68 | 7tm_1 | 7 | No | Gs |
| *ApOR63* | oki.97.9 | gbr.140.63 | 392 | 43.59 | 7tm_1 | 7 | No | Gq/11 |
