# Supplementary figures and images for: Identification of putative olfactory G-protein coupled receptors in Crown-of-Thorns starfish, Acanthaster planci
Source: BMC Genomics. 2017 May 23;18:400. doi: 10.1186/s12864-017-3793-4 (PMC5442662; doi:10.1186/s12864-017-3793-4)

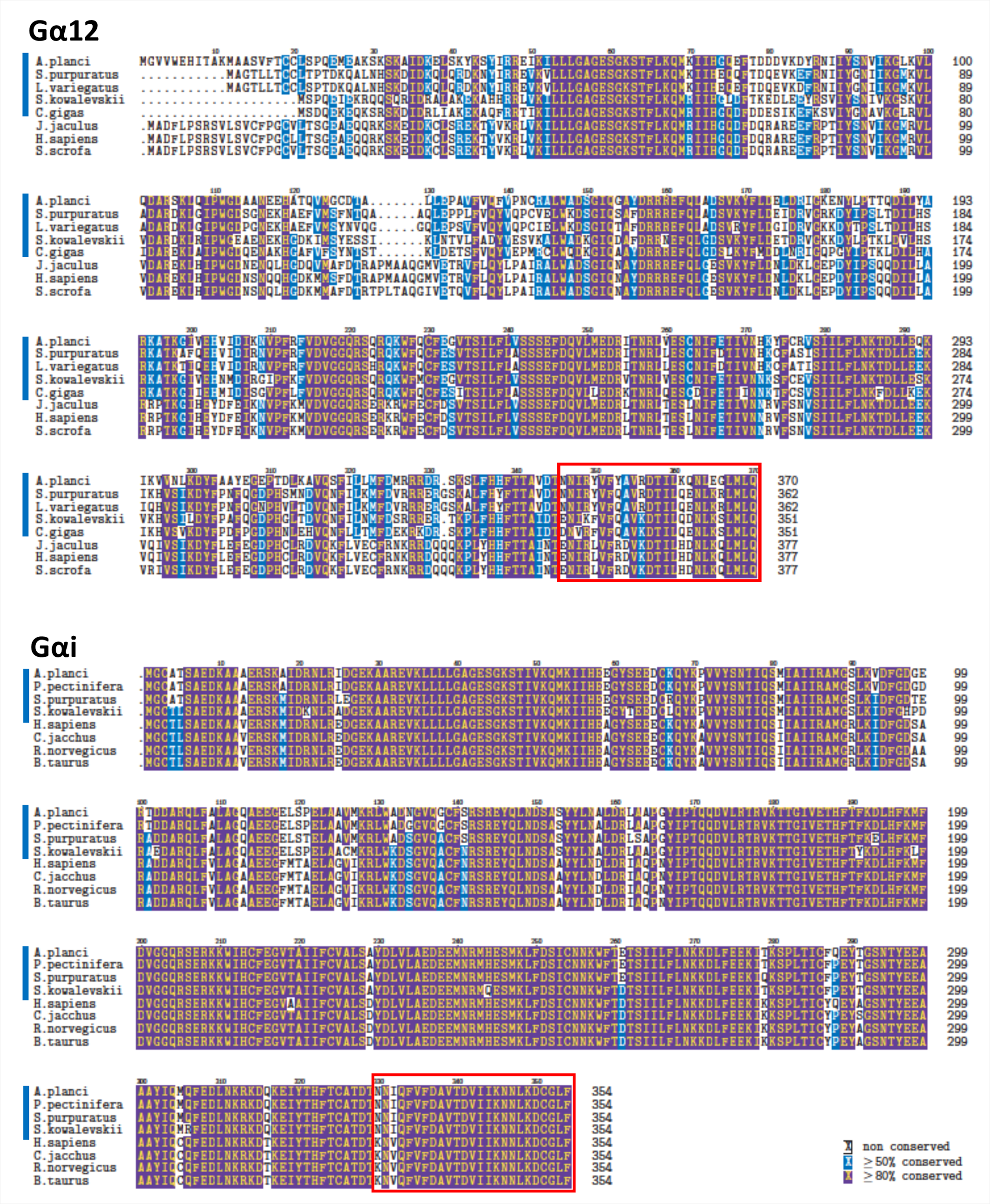

Supplement: Supplementary file 4 — Multiple sequence alignments of G α proteins from COTS and other species. Aquatic species are indicated by a blue line next to the sequence. The final 25 amino acids, to which commercially available antibodies are directed, are indicated by a red rectangle. (ZIP 21297 kb) [file 12864_2017_3793_MOESM4_ESM.zip › FigureS2a.tif]

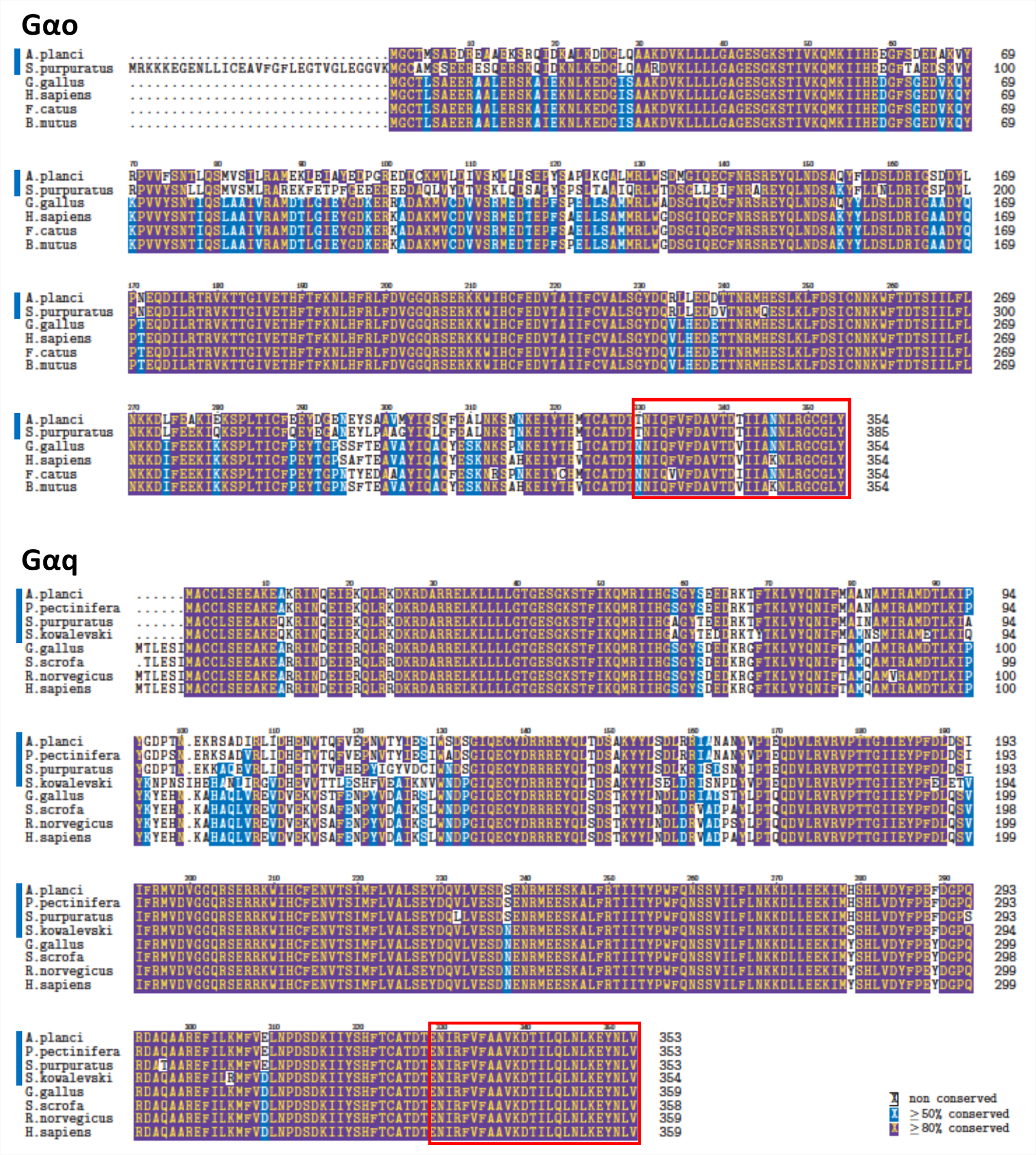

Supplement: Supplementary file 4 — Multiple sequence alignments of G α proteins from COTS and other species. Aquatic species are indicated by a blue line next to the sequence. The final 25 amino acids, to which commercially available antibodies are directed, are indicated by a red rectangle. (ZIP 21297 kb) [file 12864_2017_3793_MOESM4_ESM.zip › FigureS2b.tif]

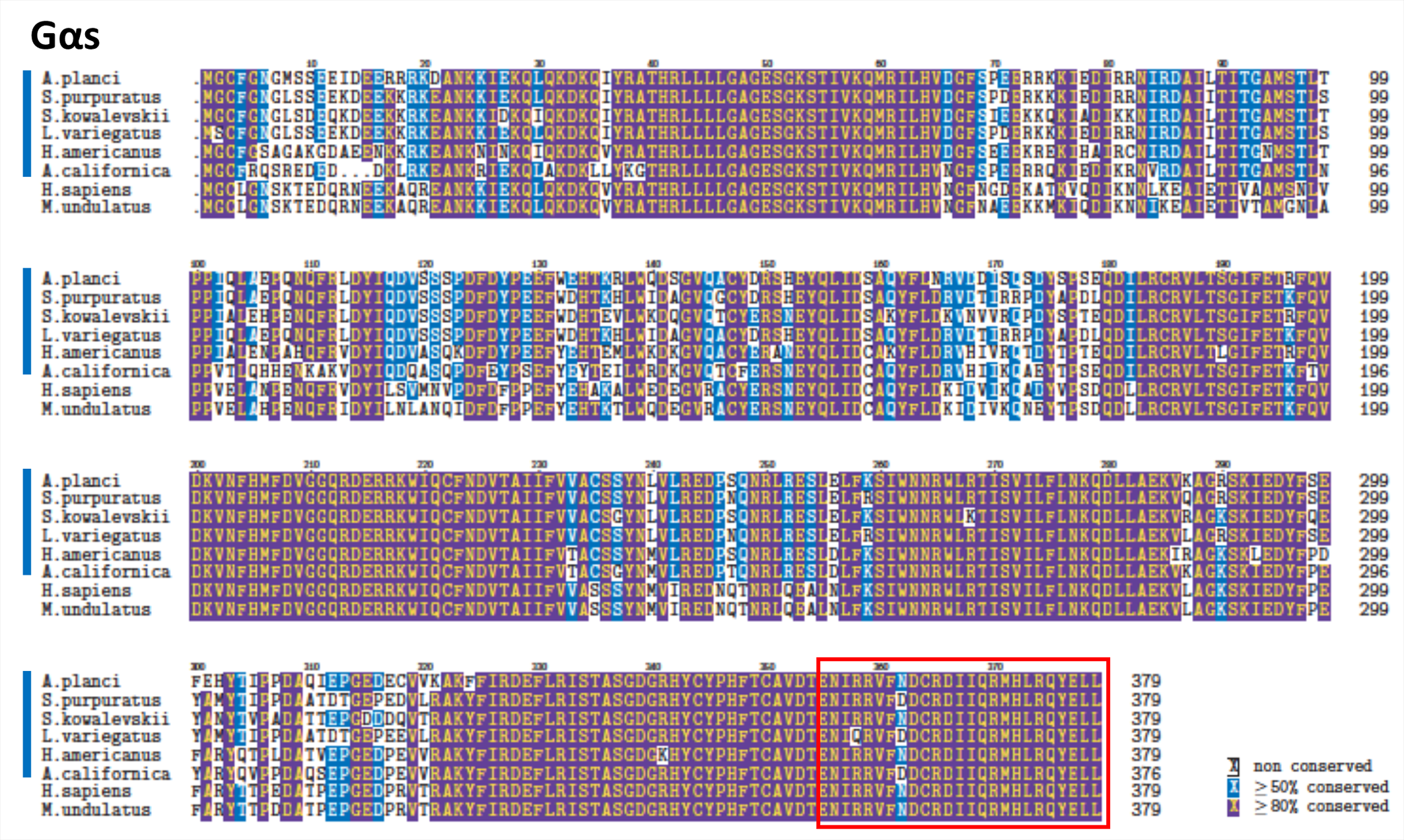

Supplement: Supplementary file 4 — Multiple sequence alignments of G α proteins from COTS and other species. Aquatic species are indicated by a blue line next to the sequence. The final 25 amino acids, to which commercially available antibodies are directed, are indicated by a red rectangle. (ZIP 21297 kb) [file 12864_2017_3793_MOESM4_ESM.zip › FigureS2c.tif]
