# Supplementary material for: Identification of putative olfactory G-protein coupled receptors in Crown-of-Thorns starfish, Acanthaster planci
Source: BMC Genomics. 2017 May 23;18:400. doi: 10.1186/s12864-017-3793-4 (PMC5442662; doi:10.1186/s12864-017-3793-4)
Supplement: Supplementary file 5 — Characterisation and expression profile of G proteins in COTS, including three uncharacterised sequences, indicating oki and gbr gene IDs, size (aa), molecular weight (kDa), Pfam domain, and expression (FPKM) in multiple COTS tissue transcriptomes. (DOCX 13 kb) [file 12864_2017_3793_MOESM5_ESM.docx]

| **Name** | **oki ID** | **gbr ID** | **Size (aa)** | **MW (kDa)** | **Pfam domain** | **podia** | **spine** | **testis** | **nerve-f** | **nerve-m** | **oocyte** | **Sensory tentacle** | **Tube foot** |
| --- | --- | --- | --- | --- | --- | --- | --- | --- | --- | --- | --- | --- | --- |
| **Gαi** | oki.164.40 | gbr.33.33 | 354 | 40.15 | G-alpha | 246.11 | 223.3 | 53.95 | 338.57 | 385.27 | 108.12 | 538.23 | 321.51 |
| **Gαs** | oki.363.3 | gbr.104.77 | 379 | 44.65 | G-alpha | 74 | 34.11 | 83.25 | 100.46 | 110.21 | 15.09 | 175 | 108.05 |
| **Gα12** | oki.62.199 | gbr.6.57 | 370 | 42.72 | G-alpha | 57.38 | 33.72 | 6.16 | 64.24 | 40.4 | 24.03 | 62.78 | 60.97 |
| **Gαq** | oki.15.66 | gbr.51.3 | 353 | 41.31 | G-alpha | 300.11 | 170.47 | 174.37 | 356.6 | 388.07 | 135.29 | 128.57 | 86.29 |
| **Gαo** | oki.266.3 | gbr.512.15 | 355 | 40.5 | G-alpha | 51.53 | 28.87 | 6.51 | 274 | 253.46 | 11.34 | 70.33 | 46.89 |
